# Pro-Inflammatory Cytokines Induce Insulin and Glucagon Double Positive Human Islet Cells That Are Resistant to Apoptosis

**DOI:** 10.3390/biom11020320

**Published:** 2021-02-19

**Authors:** Marta Tesi, Marco Bugliani, Gianmarco Ferri, Mara Suleiman, Carmela De Luca, Emanuele Bosi, Matilde Masini, Vincenzo De Tata, Conny Gysemans, Francesco Cardarelli, Miriam Cnop, Decio L. Eizirik, Piero Marchetti, Lorella Marselli

**Affiliations:** 1 Pancreatic Islet Laboratory, Department of Clinical and Experimental Medicine, University of Pisa, 56126 Pisa, Italy; marta.tesi@med.unipi.it (M.T.); m.bugliani@ao-pisa.toscana.it (M.B.); mara.suleiman@for.unipi.it (M.S.); carmela.deluca3288@gmail.com (C.D.L.); emanuele.bosi@med.unipi.it (E.B.); piero.marchetti@med.unipi.it (P.M.); 2 NEST—Scuola Normale Superiore, Istituto Nanoscienze—CNR (CNR-NANO), 56127 Pisa, Italy; gianmarco.ferri@sns.it (G.F.); francesco.cardarelli@sns.it (F.C.); 3 Department of Translational Research and New Technologies in Medicine and Surgery, University of Pisa, 56126 Pisa, Italy; matilde.masini@dps.unipi.it (M.M.); vincenzo.detata@med.unipi.it (V.D.T.); 4 Clinical and Experimental Endocrinology (CEE), Katholieke Universiteit Leuven (KU LEUVEN), 3000 Leuven, Belgium; conny.gysemans@kuleuven.be; 5 ULB Center for Diabetes Research, Medical Faculty, Université Libre de Bruxelles (ULB), 1070 Brussels, Belgium; mcnop@ulb.ac.be (M.C.); deizirik@ulb.ac.be (D.L.E.); 6 Division of Endocrinology, Erasmus Hospital, Université Libre de Bruxelles, 1070 Brussels, Belgium; 7 Indiana Biosciences Research Institute (IBRI), Indianapolis, IN 46202, USA

**Keywords:** human islets, α-cells, β-cells, insulin, glucagon, diabetes, cytokines, apoptosis

## Abstract

The presence of islet cells double positive for insulin and glucagon (Ins^+^/Glu^+^) has been described in the pancreas from both type 2 (T2D) and type 1 (T1D) diabetic subjects. We studied the role of pro-inflammatory cytokines on the occurrence, trajectory, and characteristics of Ins^+^/Glu^+^ cells in human pancreatic islets. Pancreas samples, isolated islets, and dispersed islet cells from 3 T1D and 11 non-diabetic (ND) multi-organ donors were studied by immunofluorescence, confocal microscopy, and/or electron microscopy. ND islet cells were exposed to interleukin-1β and interferon-γ for up to 120 h. In T1D islets, we confirmed an increased prevalence of Ins^+^/Glu^+^ cells. Cytokine-exposed islets showed a progressive increase of Ins^+^/Glu^+^ cells that represented around 50% of endocrine cells after 120h. Concomitantly, cells expressing insulin granules only decreased significantly over time, whereas those containing only glucagon granules remained stable. Interestingly, Ins^+^/Glu^+^ cells were less prone to cytokine-induced apoptosis than cells containing only insulin. Cytokine-exposed islets showed down-regulation of β-cell identity genes. In conclusion, pro-inflammatory cytokines induce Ins^+^/Glu^+^ cells in human islets, possibly due to a switch from a β- to a β-/α-cell phenotype. These Ins^+^/Glu^+^ cells appear to be resistant to cytokine-induced apoptosis.

## 1. Introduction

The islets of Langerhans are clusters of endocrine cells, of which 50–80% are insulin-containing β-cells, 15–30% glucagon-secreting α-cells, around 5% somatostatin-producing δ-cells, and a few % pancreatic polypeptide-releasing PP-cells that are predominantly found in the head of the pancreas [1,2,3]. A relatively small proportion of islet cells expresses more than one single hormone, and cells containing both insulin and glucagon (Ins^+^/Glu^+^ cells) have been identified. Ins^+^/Glu^+^ cells have been observed during prenatal pancreas development, representing 8–9% of the islet cell population between the 13th and 25th week of gestation [4]. In adult life, the prevalence of Ins^+^/Glu^+^ cells may increase in insulin-resistant, non-diabetic (ND) individuals that have undergone pancreatoduodenectomy [5,6], in subjects with impaired glucose tolerance, and in patients with newly diagnosed or established type 2 diabetes (T2D) [7,8]. In addition, an increased number of Ins^+^/Glu^+^ cells has been described in a small number of type 1 diabetes (T1D) subjects [9,10], which has been more recently confirmed in a larger cohort [11] and was finely studied by ultra-structural analyses [12]. 

The factors contributing to the development of Ins^+^/Glu^+^ cells in humans are still unclear, and it is unknown whether this phenomenon has a potential advantage or disadvantage in the context of severe β-cell stress. Against this background, the aim of the present study was to explore the effects of a pro-inflammatory milieu, containing interleukin-1β (IL-1β) and interferon-γ (IFN-γ), on the occurrence, trajectory and characteristics of Ins^+^/Glu^+^ cells in human pancreatic islets, and their susceptibility to apoptosis.

## 2. Materials and Methods

Human pancreatic tissue. Pancreata from 3 T1D and 3 ND donors were used for pancreatic tissue histology (Table 1). The pancreases of 8 additional ND donors were used for the isolation and study of islets (Table 1). The procedures were approved by the Ethics Committee of the University of Pisa (21 November 2013, #2615).

### 2.1. Immunohistochemistry

For immunofluorescence evaluation, human pancreatic samples were collected before islet isolation, as previously reported [13]. Four-micrometer-sections of pancreatic samples from organ donors were stained for insulin and glucagon by 1 h incubation at room temperature with, respectively, polyclonal guinea pig anti-insulin antibody (Abcam, Cambridge, UK) at 1:100 dilution, and monoclonal mouse anti-glucagon antibody (Sigma-Aldrich, St. Louis, MO, USA) at 1:3000 dilution. Insulin and glucagon detection was realized by 1 h incubation at room temperature with, respectively, Alexa Fluor 594-conjugated donkey anti-guinea pig (Jackson ImmunoResearch, Baltimore, PA, USA) secondary antibody at 1:200 dilution and Alexa Fluor® 488 Donkey anti-mouse (Jackson ImmunoResearch) secondary antibody at 1:200 dilution. Fluorescence images were acquired using a Leica fluorescent microscope equipped with Leica MetaMorph® software, v1.8.0. 

### 2.2. Human Islet Isolation and Culture 

Islets were isolated by collagenase digestion followed by density gradient purification, as previously reported [14,15]. Maintenance culture was at 37 °C and 5% CO_2_ atmosphere, in M199 culture medium complemented with 10% bovine serum, 100 U/mL penicillin, 100 μg/mL streptomycin, 750 ng/mL amphotericin B, and 50 μg/mL gentamicin. Within 3 days from isolation, islets were exposed to 50 U/mL IL-1β and 1000 U/mL IFN-γ (Sigma-Adrich) for up to 120 h. In a subset of experiments, islets were exposed to the cytokines for 48 h; then, the islets were washed and cultured in control medium for additional 48 h.

### 2.3. Electron Microscopy

To perform anti-insulin and anti-glucagon immunogold labeling [16], ultrathin pancreatic sections mounted on nickel grids were placed on droplets of freshly prepared 1% aqueous periodic acid for 8 min at room temperature and rinsed with distilled water. Sections were conditioned with phosphate-buffered saline (PBS:0.01 M phosphate buffer, pH 7.2, 0.15 M NaCl) containing 1% bovine serum albumin (BSA), 0.01% Triton X-100, and 0.01% Tween 20, transferred to polyclonal guinea pig anti-insulin antibody (Abcam) at 1:100 dilution, or to monoclonal mouse anti-glucagon antibody (Sigma-Aldrich) at 1:3000 dilution, and incubated for 1 h at room temperature. Then, grids were incubated with 1:10 diluted protein A-gold complex (15 nm gold particles) (Agar Scientific, Stansted, UK) for 1 h. Finally, sections were contrasted with uranyl acetate and lead citrate prior to examination with a 902 Zeiss electron microscope. In negative control incubations, the primary antibody was omitted. Volume densities of insulin and glucagon granules were assessed as previously described [12,14]. Briefly, a graticule (11 × 11 cm), composed of 169 points, was used to overlay micrographs, acquired at X10,000. Volume density was calculated as Pi/Pt, where Pi is the number of points within the sub-cellular component and Pt is the total number of points and expressed in milliliters/100 mL tissue (mL%). 

### 2.4. Apoptosis

In electron microscopy analysis, islet cell apoptosis was detected by the presence of marked chromatin condensation and/or blebs and quantified as previously reported [12]. Apoptosis was also assessed in dispersed islet cells by Terminal deoxynucleotidyl transferase dUTP nick end labeling (TUNEL) assay. In brief, islets were dispersed with Accutase (Sigma-Aldrich) and plated on sterile 35 mm, high walls, #1.5 polymer coverslips (Ibidi, Martinsried, Germany), suitable for confocal microscopy, coated with of the extracellular matrix components from Engelbreth–Holm–Swarm mouse sarcoma (Sigma-Aldrich) as detailed elsewhere [17]. After 48 h exposure to cytokines, apoptotic islet cells were evaluated by the in situ Cell Death Detection Kit, Fluorescein (Sigma-Aldrich) following the manufacturer’s protocol. Co-localization experiments were performed using a mouse monoclonal anti-insulin antibody (Bio-Rad, Oxford, UK) and a rabbit polyclonal anti-glucagon antibody (DAKO, Glostrup, Denmark), and Alexa Fluor 647-conjugated donkey anti-mouse antibody (Invitrogen, Carlsbad, CA, USA) and Alexa Fluor 555-conjugated donkey anti-rabbit antibody (Invitrogen) as secondary antibodies. Images were acquired with a laser scanning confocal microscope Zeiss LSM 800 (with Airyscan) equipped with two GaAsP detectors and analyzed with ImageJ software (version 1.51, https://imagej.nih.gov/ij/index.html (accessed on 15 February 2021)).

### 2.5. Gene Expression

As previously detailed [13,14,16,17,18], total RNA was extracted using the PureLink^TM^ RNA Mini kit (Life technologies, Carlsbad, CA, USA) and quantified by absorbance at A260/A280 nm (ratio >1.95) in a Nanodrop 2000c spectrophotometer (Thermo Scientific, Waltham, MA, USA). For quantitative RT-PCR experiments, 1.5 μg total RNA was reverse-transcribed with SuperScript VILO Master Mix (Life technologies). Messenger RNA levels of genes of interest were amplified, quantified, and normalized for the reference gene beta actin in a VIIA7 instrument (Life technologies). 

### 2.6. Statistical Analysis

Data are expressed as mean ± SE. The two-tailed Student’s *t* test was used to assess differences between two groups. For three or more groups, ANOVA was used followed by the Bonferroni correction. A *p* value less than 0.05 was considered statistically significant.

## 3. Results

### 3.1. Ins^+^/Glu^+^ Cells Are Present in Pancreatic Islets of T1D Donors

**Figure 1:**
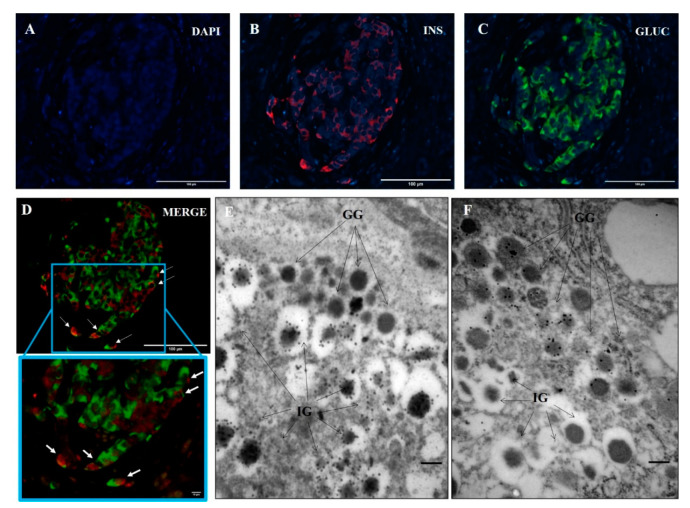
Representative images showing insulin and glucagon double positive (Ins^+^/Glu^+^) cells in human type 1 diabetes (T1D) pancreatic islets. Fluorescence microscopy images of DAPI (**A**), DAPI/insulin (**B**) and DAPI/glucagon (**C**) immunostainings are shown, with a few cells containing both insulin and glucagon positivity indicated in (**D**) (scale bar in A-D corresponds to 100 µm); electron microscopy images of insulin (**E**) and glucagon (**F**) immunogold staining (scale bar corresponds to 0.26 µm). IG: insulin granules; GG: glucagon granules.

To assess the occurrence of cells double positive for insulin and glucagon in T1D pancreas, we used fluorescence light microscopy (Figure 1A–D) and electron microscopy (Figure 1E,F). As shown in Figure 1, in some cells, insulin (panel B) and glucagon (panel C) staining co-localized (merge is shown in panel D, with a magnification of an area of the panel also reported). Quantification of the different cell types was not performed in this set of experiments. Electron microscopy showed the presence of cells containing both insulin and glucagon granules, as determined by the typical ultrastructural appearance of these granules [12,19,20] and insulin or glucagon gold immunostaining (Figure 1E,F). The proportion of Ins^+^/Glu^+^ cells was higher in T1D islets (#1: 12% of 150 endocrine cells counted; #2: 13% of 151 endocrine cells) than in ND islets (#1: 0% of 162 endocrine cells; #2: 3% of 217 endocrine cells; #3: 1% of 159 endocrine cells). 

### 3.2. Pro-Inflammatory Cytokines Induce Ins^+^/Glu^+^ Cells 

**Figure 2:**
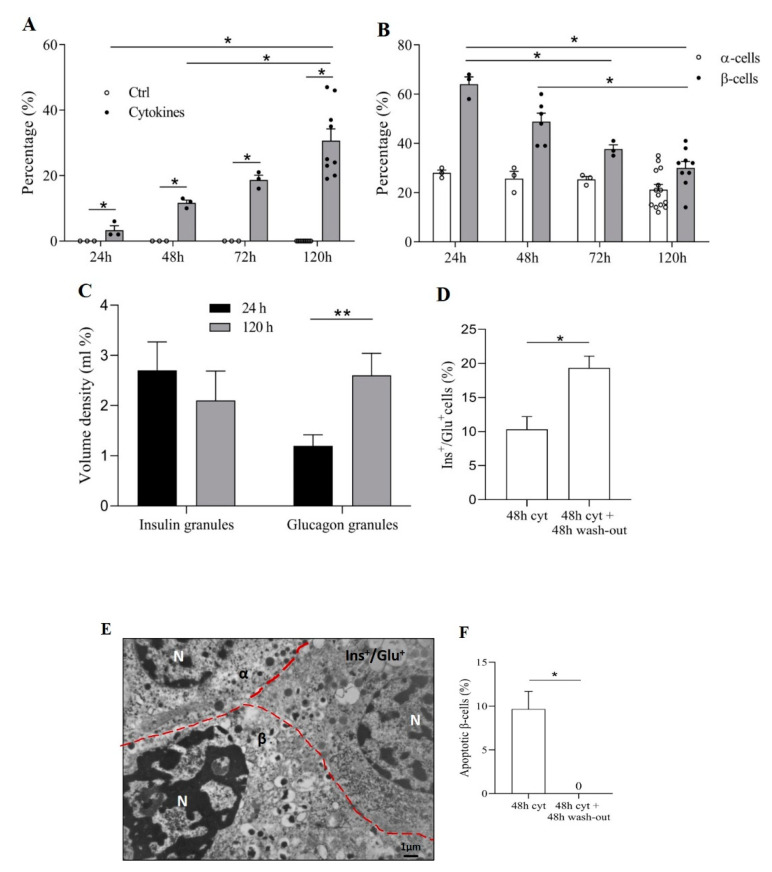
Pro-inflammatory cytokines induce Ins^+^/Glu^+^ cells in non-diabetic (ND) human islets. (**A**) Percentage of Ins^+^/Glu^+^ cells at different time points of exposure to pro-inflammatory cytokines compared to control. (**B**) Percentage of α- and β-cells at different time points of exposure to pro-inflammatory cytokines. (**C**) Volume density of insulin and glucagon granules in Ins^+^/Glu^+^ cells after 24 h and 120 h cytokine exposure. (**D**) Percentage of Ins^+^/Glu^+^ cells after 48 h cytokine exposure, followed or not by 48 h wash-out (medium without cytokines). (**E**) Electron microscopy image showing an apoptotic β-cell (β) with chromatin condensation in the nucleus (N); in the same picture a normal α-cell (α) and an Ins^+^/Glu^+^ cell (Ins^+^/Glu^+^) are depicted (scale bar corresponds to 1 µm). (**F**) Percentage of apoptotic β-cells after 48 h cytokine exposure followed or not by 48 h wash-out (medium without cytokines). Percentages assessed by electron microscopy are relative to total endocrine cell number. * *p* < 0.05, ** *p* < 0.01. Percentages assessed by electron microscopy are relative to total endocrine cell number. * *p* < 0.05, ** *p* < 0.01.

To test whether pro-inflammatory cytokines affect the occurrence of cells containing both insulin and glucagon, isolated human islets were exposed to IL-1β (50 U/mL) and IFN-γ (1000 U/mL) for up to 120 h. Electron microscopy showed a significant and progressive increase of the proportion of Ins^+^/Glu^+^ cells (from 3 ± 1% (124 cells counted) at 24 h to 31 ± 4% (715 cells counted) at 120 h), whereas no change was seen in non-treated islets (Figure 2A). This was associated with a progressive reduction of β-cell percentage (from 64 ± 3% at 24 h to 30 ± 3% at 120 h) in cytokine-exposed islets (Figure 2B). No significant change was observed in α-cell proportion (from 28 ± 1% at 24 h to 21 ± 2% at 120 h) (Figure 2B). As shown in Figure 2C, in Ins^+^/Glu^+^ cells the volume density of insulin granules tended to decrease (approximately −25%) after 120 h vs. 24 h cytokine exposure, and glucagon granule volume density increased significantly (approximately twofold).

We then assessed whether the occurrence of Ins^+^/Glu^+^ cells was a reversible phenomenon. To do so, human islets were exposed to the cytokine combination for 48 h. Then, they were washed and cultured for additional 48h in control medium. After the 48 h exposure to cytokines, the proportion of cells containing both insulin and glucagon granules was 10 ± 2% (out of 150 cells counted). After the removal of the pro-inflammatory milieu and the additional 48 h culture in control medium, such a proportion increased to 19 ± 2% (out of 126 cells, *p* < 0.05) (Figure 2D). Electron microscopy allowed the identification and quantification of β-cells with ultrastructural signs of apoptosis (Figure 2E). The percentage of such cells decreased markedly after cytokine wash-out (from 10 ± 3% to 0%, *p* < 0.05) (Figure 2F).

### 3.3. Ins^+^/Glu^+^ Cells Are Resistant to Apoptosis

**Figure 3:**
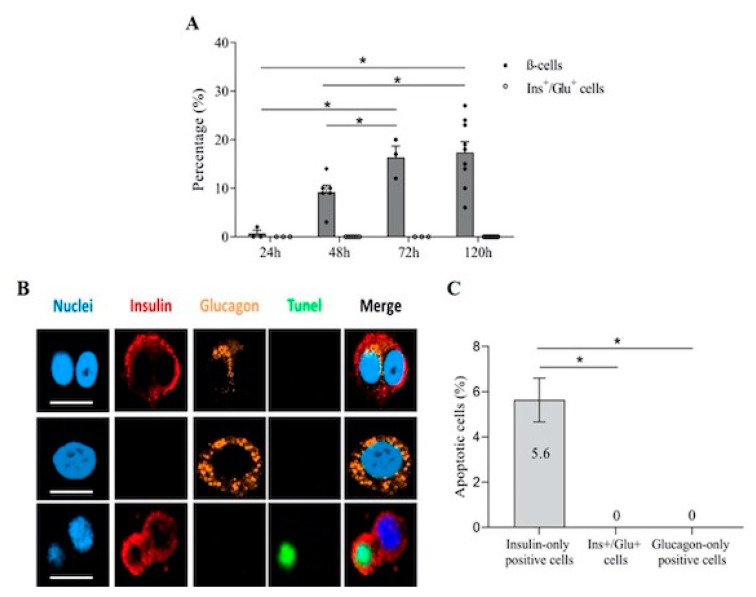
Pro-inflammatory cytokines induce apoptosis in β- but not in Ins^+^/Glu^+^ cells. (**A**) Percentage of β- and Ins^+^/Glu^+^ cells with signs of apoptosis in whole human islets at different time points of pro-inflammatory cytokine exposure, quantified by electron microscopy. (**B**) Representative images of confocal microscopy analysis of dispersed human islet cells, stained for DAPI (nucleus, blue), insulin (red), glucagon (yellow) and TUNEL (apoptotic nucleus, green), after 48 h cytokine exposure (scale bar corresponds to 10 µm). (**C**) Percentage of insulin-only positive cells, Ins^+^/Glu^+^ cells and glucagon-only positive cells stained by TUNEL. Percentages are relative to total endocrine cell number. * *p* < 0.05.

Cytokine exposure induced a marked, progressive increase in the percentage of apoptotic cells containing insulin granules only (Figure 3A), as assessed by electron microscopy. Intriguingly, Ins^+^/Glu^+^ cells showed no or minimal signs of apoptosis (Figure 3A). To assess whether cells containing both insulin and glucagon granules are protected from apoptosis, experiments were performed with single cells obtained by enzymatic dissociation of human islets. Immunofluorescent staining identified cells containing only insulin (*n*: 184), only glucagon (*n*: 88) or both insulin and glucagon (*n*: 40) (Figure 3B). Apoptosis was evaluated by TUNEL staining (Figure 3B). Notably, insulin-only positive cells showed an apoptotic rate of 5.6 ± 1.0%, whereas none of the Ins^+^/Glu^+^ cells stained for TUNEL (Figure 3C). In addition, no glucagon-only containing cells showed signs of TUNEL positivity (Figure 3C).

### 3.4. Cytokines Affect β-Cell Identity

**Figure 4:**
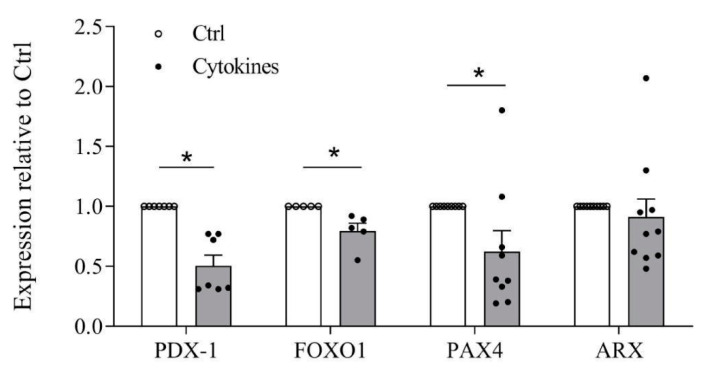
*PDX-1*, *FOXO1*, *PAX4*, and *ARX* gene expression (relative to control) assessed by RT-PCR after 48 h exposure to cytokines. * *p* < 0.05.

In order to investigate whether the induction of Ins^+^/Glu^+^ cells was associated with an alteration of islet cell identity, the expression of key islet cell genes was assessed. As shown in Figure 4, 48-h cytokine exposure down-regulated genes that mark and preserve β-cell identity, such as *PDX-1, FOXO1* and *PAX4*, whereas no change occurred in the expression of *ARX*, a marker of α-cell identity. These changes suggest a maintenance of the α-cell phenotype and a loss of β-cell phenotype, under the stressful effects of pro-inflammatory cytokines.

## 4. Discussion

Human islets in T1D contain Ins^+^/Glu^+^ cells. Here, we show the key role of pro-inflammatory cytokines in the occurrence of Ins^+^/Glu^+^ cells in human pancreatic islets. These cells seem to derive from β-cells and the change into bi-hormonal cells confers resistance against cytokine-induced apoptosis, suggesting a protective adaptation. 

The presence of Ins^+^/Glu^+^ cells has been described in pancreatic islets of subjects with T1D in several, but not all, recently published studies using different techniques [9,10,11,12,20]. In our limited number of cases (3 ND and 2–3 T1D subjects), we confirm an increased proportion of this bi-hormonal cell type in T1D. Interestingly, Ins^+^/Glu^+^ cells have also been observed in pre-diabetic NOD (non-obese diabetic) mice, a murine model of T1D (C.G., unpublished data). 

In experiments with isolated human islets, we explored the direct impact of pro-inflammatory cytokines that probably contribute to β-cell death in T1D [21] on the occurrence and trajectory of Ins^+^/Glu^+^ cells. We showed that IL-1β and IFN-γ induced Ins^+^/Glu^+^ cells, the proportion of which increased with the length of exposure. Other pro-inflammatory conditions may exert similar actions. Moin et al. showed that the islets of subjects with chronic pancreatitis exhibit higher proportions of Ins^+^/Glu^+^ cells [22].

Under our experimental conditions, Ins^+^/Glu^+^ cells seem to derive from β-cells as suggested by the observation that cytokine exposure progressively decreases human islet β-cell numbers (containing insulin granules only); after 120 h exposure the β-cell proportion is halved. This reduction can only partially be attributed to β-cell loss due to apoptosis, which was around 20% at 120 h. The proportion of α-cells (containing only glucagon granules) remained fairly constant over the same period of time. In further support of the β-cell origin of the bi-hormonal cells, we found that cytokines significantly reduced the expression of *PDX-1, FOXO1*, and *PAX4,* transcription factors known to be involved in the maintenance of β-cell identity [23,24,25,26,27]. In our experiments, we did not measure the expression of additional markers of β-cell de-differentiation [23,24,25,26,27]. Previous work has shown more or less marked reduction of the expression of β-cell identity markers such as *PDX-1*, *FOXO1, MAFA, NKX6.1, PAX6*, and *SLC2A2* in isolated human islets after 48 h exposure to IL-1β and IFN-γ [28] or 24 h exposure to IL-1β [29]. However, other genes that are associated with early pancreatic development and/or successive cell differentiation (such as *POU5F1*, *SOX17*, *NEUROD1*) have been reported not to be affected by cytokines [28,30].

It remains to be determined whether these changes prelude human β- to α-cell trans-differentiation. This phenomenon has been reported in rodent models [31,32,33], but it is difficult to be confirmed in human islets for lack of proper lineage tracing tools. Spijker et al. have shown that at least some primary human β-cells can convert into α-cells ex vivo, as assessed by lentivirus-mediated β-cell lineage tracing [34]. The trans-differentiated α-cells were ultrastructurally identical to native α-cells. The transition of β- into α-cells occurred after β-cell degranulation and was characterized by the continued presence of β-cell specific transcription factors *PDX-1* and *NKX6.1* in glucagon-positive cells. *ARX* knockdown inhibited the trans-differentiation. This last observation is in keeping with our finding that *ARX* expression was not affected by cytokines. In addition, other α-cell transcription factors previously identified by single-cell RNAseq analysis (such as *IRX2*, *FEV*, and *SMARCA1*) were found not to change upon cytokine exposure [28,30].

Using electron microscopy in whole islets and confocal microscopy in dispersed islet cells, we observed cytokine-induced apoptosis in insulin-only containing cells, whereas Ins^+^/Glu^+^ cells did not show any signs of apoptosis. This intriguing finding suggests that a mixed β/α-cell phenotype might provide resistance to the cytokine insult. A recent study showed that β-cell specific manipulation of the unfolded protein response in NOD mice induced transient β-cell dedifferentiation, which resulted in reduced β-cell apoptosis [35]. Stepwise incubations of the murine β-TC3 cell line with cytokines selected cells resistant to cytokine toxicity, while there was loss of insulin secretion [36]. α-cells are more resistant than β-cells to both viral infection [37] and metabolic stress [38], reinforcing the concept that β-cells that acquire α-cell characteristics may be better equipped to endure diabetogenic stresses. The functional and molecular characterization of human islet single cells [39,40,41,42], including those co-expressing more than one hormone, will provide further insights into these issues.

In summary, this study confirms the increased presence of Ins^+^/Glu^+^ double-positive cells in human T1D pancreatic islets. Using human islets ex vivo, we demonstrate that pro-inflammatory cytokine exposure induces Ins^+^/Glu^+^ cells in a time-dependent manner. This cell type may derive from β-cells and appears to be less prone to cytokine-induced apoptosis, as compared to cells containing only insulin. These changes could represent an adaptive behavior to survive in a hostile environment and confer potential evolutionary advantage to β cells. It remains to be determined whether these double positive cells can produce and release insulin at levels comparable with those from insulin-only containing cells and eventually regain an Ins^+^ only phenotype, once the inflammatory stress is resolved.

## Figures and Tables

**Table 1:** Clinical characteristics of the donors.

	Age (y)	Sex	BMI (kg/m^2^)	Duration of Diabetes (y)	Anti-GAD Positivity	Cause of Death	Use in the Study
T1D #1	39	M	25.1	23	Yes	CVD	Pancreas histology
T1D #2	24	M	25.7	2	Yes	Trauma	Pancreas histology
T1D #3	39	F	24.5	21	NA	Trauma	Pancreas histology
ND #1	38	F	22.5	-	NA	CVD	Pancreas histology
ND #2	22	M	19.6	-	NA	Trauma	Pancreas histology
ND #3	73	M	24.2	-	NA	CVD	Pancreas histology
ND #4	75	M	27.7	-	NA	CVD	Isolated islets
ND #5	38	F	22.5	-	NA	CVD	Isolated islets
ND #6	64	F	24.2	-	NA	CVD	Isolated islets
ND #7	70	M	20.4	-	NA	CVD	Isolated islets
ND #8	56	F	22.5	-	NA	CVD	Isolated islets
ND #9	85	F	20.8	-	NA	Trauma	Isolated islets
ND #10	60	M	22.9	-	NA	CVD	Isolated islets
ND #11	69	F	33.1	-	NA	CVD	Isolated islets

BMI = body mass index, CVD = cardiovascular disease, anti-GAD= anti-glutamic acid decarboxylase antibody, NA= not available.

## Data Availability

All findings and conclusions are based on the presented figures in the text. Original source files can be sent from the corresponding author, Lorella Marselli, upon request.
